# International chemical identifier for chemical reactions

**DOI:** 10.1186/1758-2946-5-S1-O16

**Published:** 2013-03-22

**Authors:** Guenter Grethe, Jonathan Goodman, Chad Allen

**Affiliations:** 1 352 Channing Way, Alameda, CA 94502-7409, USA; 2 Department of Chemistry, University of Cambridge, Cambridge, CB2 1EW, UK

## 

An open-access software for creating a unique, text-based identifier for reactions (RInChI) was developed by the Goodman group at the University of Cambridge, based on the IUPAC International Chemical Identifier (InChI) standard. RInChIs describe the substances (reactants, products, reagents and solvents) participating in a reaction with their respective InChIs. The structure of RInChIs is analogous to that of InChIs. In addition to generate RInChIs from widely used Rxnfiles and RDfiles, the software also includes the generation of long- and short-form, hashed representations - RInChIKeys. Furthermore, the software allows to reversibly convert between CT-files and RInChIs, to search for specific substances and their specific roles in reactions and to analyze databases. All these functions are available through web-based tools. An easy-to-use website has been developed at the University of Cambridge and is freely accessible at http://www-rinchi.ch.cam.ac.uk/. We will discuss details of the program and the status of the RInChI project.

